# Biomechanical characteristics of Sanders type II and III calcaneal fractures fixed by open reduction and internal fixation and percutaneous minimally invasive fixation

**DOI:** 10.1186/s13018-024-04606-1

**Published:** 2024-03-05

**Authors:** Wu Ren, Kailu Zhang, Ziya Zhao, Xueling Zhang, Fei Lin, Yawei Li, Ke Bao, Jun Yang, Jinlong Chang, Jia Li

**Affiliations:** 1 The First Affiliated Hospital of Xinxiang Medical University, School of Medical Engineering, Xinxiang Medical University, Xinxiang, 453003 Henan China; 2 Engineering Technology Research Center of Neurosense and Control of Henan Province, Xinxiang Engineering Technology Research Center of Intelligent Rehabilitation Equipment, Xinxiang, 453003 Henan China; 3 Hunan Normal University, Changsha, 410000 Hunan China

**Keywords:** Calcaneal fracture, Biomechanics, Sander classification, Internal fixation, Finite element analysis

## Abstract

**Background:**

This work investigated the differences in the biomechanical properties of open reduction and internal fixation (ORIF) and percutaneous minimally invasive fixation (PMIF) for the fixation of calcaneal fractures (Sanders type II and III calcaneal fractures as examples) through finite element analysis.

**Methods:**

Based on CT images of the human foot and ankle, according to the principle of three-point fixation, namely the sustentaculum tali, the anterior process and the calcaneal tuberosity were fixed. Three-dimensional finite element models of Sanders type II and III calcaneal fractures fixed by ORIF and PMIF were established. The proximal surfaces of the tibia, fibula and soft tissue were constrained, and ground reaction force and Achilles tendon force loads were added to simulate balanced standing.

**Results:**

The maximum stress was 80.54, 211.59 and 113.88 MPa for the calcaneus, screws and plates in the ORIF group and 70.02 and 209.46 MPa for the calcaneus and screws in the PMIF group, respectively; the maximum displacement was 0.26, 0.21 and 0.12 mm for the calcaneus, screws and plates in the ORIF group and 0.20 and 0.14 mm for the calcaneus and screws in the PMIF group, respectively. The values obtained from the simulation were within the permissible stress and elastic deformation range of the materials used in the model, and there was no significant stress concentration. The maximum stress and displacement of the calcaneus and implants were slightly lower in the PMIF group than in the ORIF group when fixing Sanders type II and III calcaneal fractures.

**Conclusions:**

This study may provide a reference for optimising the design of implants, the development of individualised preoperative plans and the choice of clinical surgical approach.

## Introduction

Calcaneal fractures account for 2% of all body fractures and 60% of foot fractures, 75% of which are displaced intra-articular fractures [1]. Calcaneal fractures are usually caused by an impact on the calcaneus during a fall from a height. High energy impact on the calcaneus causes collapse of the posterior talar articular surface [2]. The calcaneal trabeculae are arranged in the direction of the tension and pressure to which they are subjected, including the tension trabeculae and the pressure trabeculae. The pressure trabeculae are dense below the posterior talar articular surface [3]. Due to the specific anatomy of the calcaneus, the main weight-bearing area is the posterior talar articular surface, and this is where most calcaneal fractures occur [4]. Calcaneal fractures are mainly manifested by heel tenderness and the inability to walk on the ground, which affects people’s health and life. And the best way to treat calcaneal fractures is still unclear [2, 5].

Most clinical classifications of calcaneal fractures use the Sanders classification proposed by Sanders [6] in 1990. This classification mainly reflects the degree of injury to the posterior talar articular surface of the calcaneus and has important implications for the clinical treatment and prognosis of calcaneal fractures. Depending on the degree of fracture and soft tissue damage, treatment options for calcaneal fractures include non-surgical and surgical treatments [7, 8]. Non-surgical treatment, also known as conservative treatment, is indicated for patients with comminuted calcaneal fractures, poor soft tissue conditions or other serious diseases. The treatment does not reset the fracture sites but allows for immobilisation in a cast and early weight bearing. The pain and swelling of the patient’s foot are reduced by applying ice, simple activities and appropriate functional exercises. Surgical treatment options include open reduction and internal fixation (ORIF) and percutaneous minimally invasive fixation (PMIF).

ORIF is suitable for displaced calcaneal fractures, particularly intra-articular fractures of the calcaneus, and is effective in restoring the force line bearing a heavy burden on the hindfoot [9]. This treatment achieves good results in terms of reliable fixation and accurate restoration through the use of implants such as plates and screws, with a cure rate of over 75% [10]. Surgical treatment aims to restore the geometry of the calcaneus and the flatness of the articular surface. The fracture blocks can be repositioned to accurately restore the posterior talar articular surface and the anatomical relationship between the three subtalar articular surfaces, the degree of which is closely related to the clinical efficacy of the calcaneal fracture [11]. ORIF requires extensive dissection of the soft tissue to fully expose the fracture, and the soft tissue incision margins may become infected or even necrotic after surgery, which is more damaging to the body [12]. PMIF is indicated for mild calcaneal fractures with partial loss of calcaneal height and mild widening, which can be repositioned with the aid of steel nails and Kirschner wires. This method does not require extensive soft tissue dissection, avoids soft tissue necrosis, reduces intraoperative bleeding and reduces the risk of post-operative complications [13]. The treatment of displaced intra-articular calcaneal fractures continues to be a challenge [14–16]. Research showed that conventional ORIF is more effective but has a higher complication rate of 11.7% to 35%. In recent years, PMIF technology has significantly reduced the post-operative complication rate to 0–13% [17].

In recent years, with the continuous development of computer technology, biomechanics, medicine, and physics, finite element simulation techniques have been increasingly used in areas such as fracture fixation. Hanbin Ouyang [18] designed a calcaneal locking plate with good biomechanical properties by topology optimisation and compared it with a conventional locking plate using the finite element method. The new design was found to have a more reasonable stress distribution and demonstrated superiority in fatigue strength. Ming Ni [19] used finite element software to simulate the biomechanical properties of calcaneal fractures fixed with absorbable screws and to calculate the stress and displacement distribution of the calcaneus and implants. The results showed that the stress and displacement in the calcaneus were within acceptable limits, which was similar to the results of Haowei Zhang [20]. Ching-Hsuan Chen [21] analysed the use of the whole locking, nonlocking, and hybrid screws to fix Sanders type II calcaneal fractures and found that hybrid screws had a biomechanical advantage over whole locking screws. Qing-Jiang Pang [22] took a finite element approach to investigate the need for talus support screws in the fixation of calcaneal fractures and found that they were key to achieving biomechanical stability. Several clinical surgeries have demonstrated that minimally invasive screws can accurately restore the anatomical structure of the calcaneus and achieve a good prognosis [23, 24]. In most of these, the traction of the ligaments, plantar fascia, and soft tissue on the bones was not taken into account. Ming Zhang and Duo Wai-Chi Wong [25] developed a methodological quality assessment instrument for single-subject finite element analysis (MQSSFE) in computational orthopaedics. The instrument contained 37 items with 6 domains such as model reconstruction and configuration, model verification, and model assumption and validity. The MQSSFE was compared with the modified Down and Black instrument [26] to demonstrate that the MQSSFE was valid and reliable.

The calcaneus and the implants have complex mechanical behaviour, both in the early stages of surgery and in the post-operative healing period. This is due to a combination of the geometry of the bones, the fracture site, and the configuration of the implants. Traditional open reduction surgery is more effective but has a higher complication rate. Because of the development of minimally invasive techniques, calcaneal fracture complications can be effectively reduced. However, due to the complexity of calcaneal fracture, the surgeons need to choose the appropriate treatment plan according to the characteristics of patients and specific conditions, so as to achieve better post-operative efficacy [27].

In summary, this work compared the biomechanical properties of the ORIF and PMIF surgical approaches to fixing calcaneal fractures using finite element analysis (FEA). The aim was to investigate the differences in biomechanical properties. This study: (1) the disadvantage of the irreversibility of clinical surgery was overcome, experimental consumables were reduced and costs were saved to a certain extent, the simulation parameters can be optimised several times through clinical feedback, and the results obtained are useful for exploring the pathogenesis of human orthopaedic diseases and for refining models; (2) research on the mechanics of the osteomuscular system, the development of implants and other orthopaedic rehabilitation devices and surgical optimisation are assisted; and (3) through parametric mechanical simulations, clinicians are provided with references to design and refine treatment plans and rehabilitation programmes.

## Materials and methods

### Geometry reconstruction

**Fig. 1:**
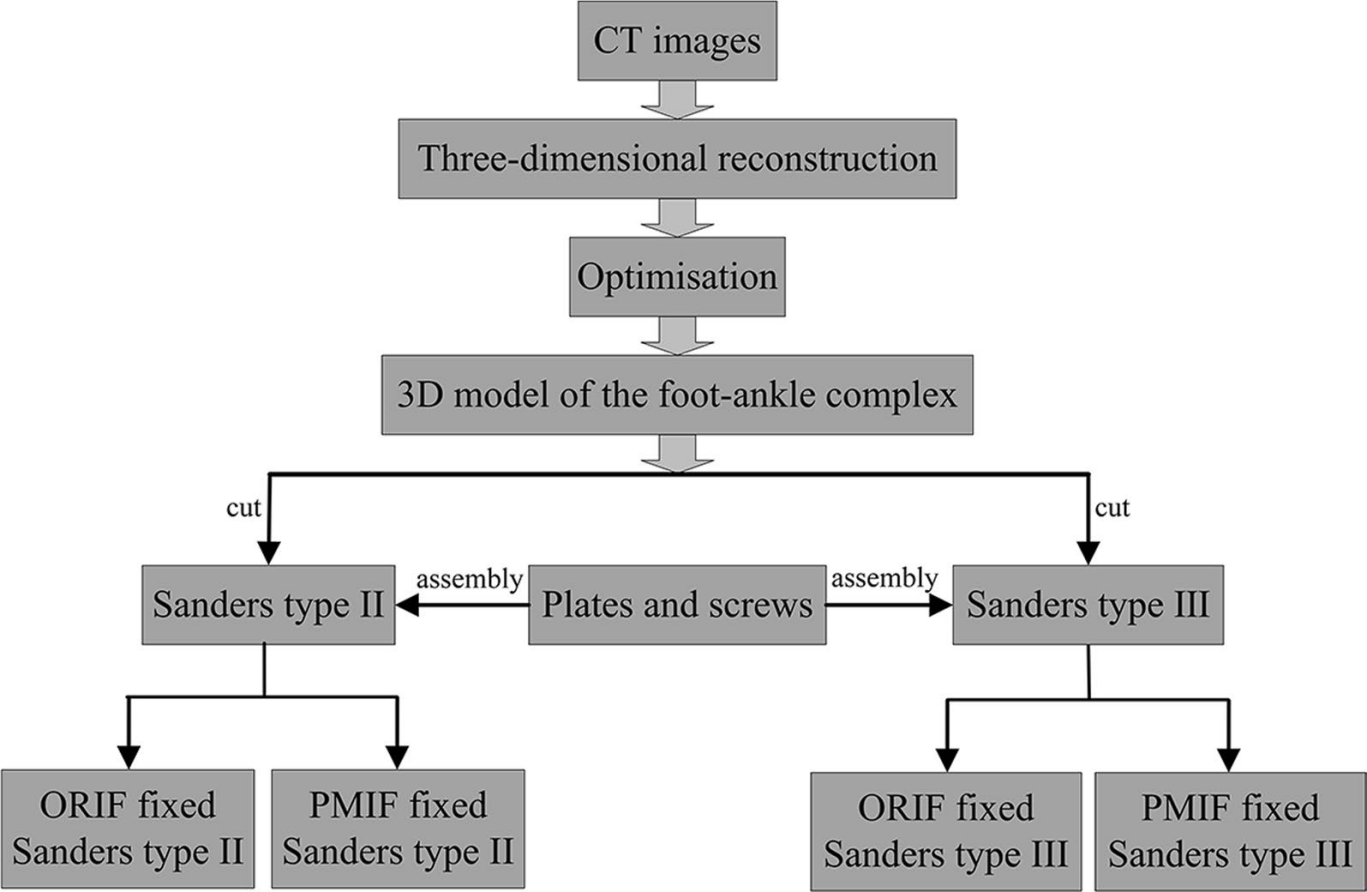
Modelling steps for the fixation of Sanders type II and III calcaneal fractures by ORIF and PMIF

**Fig. 2:**
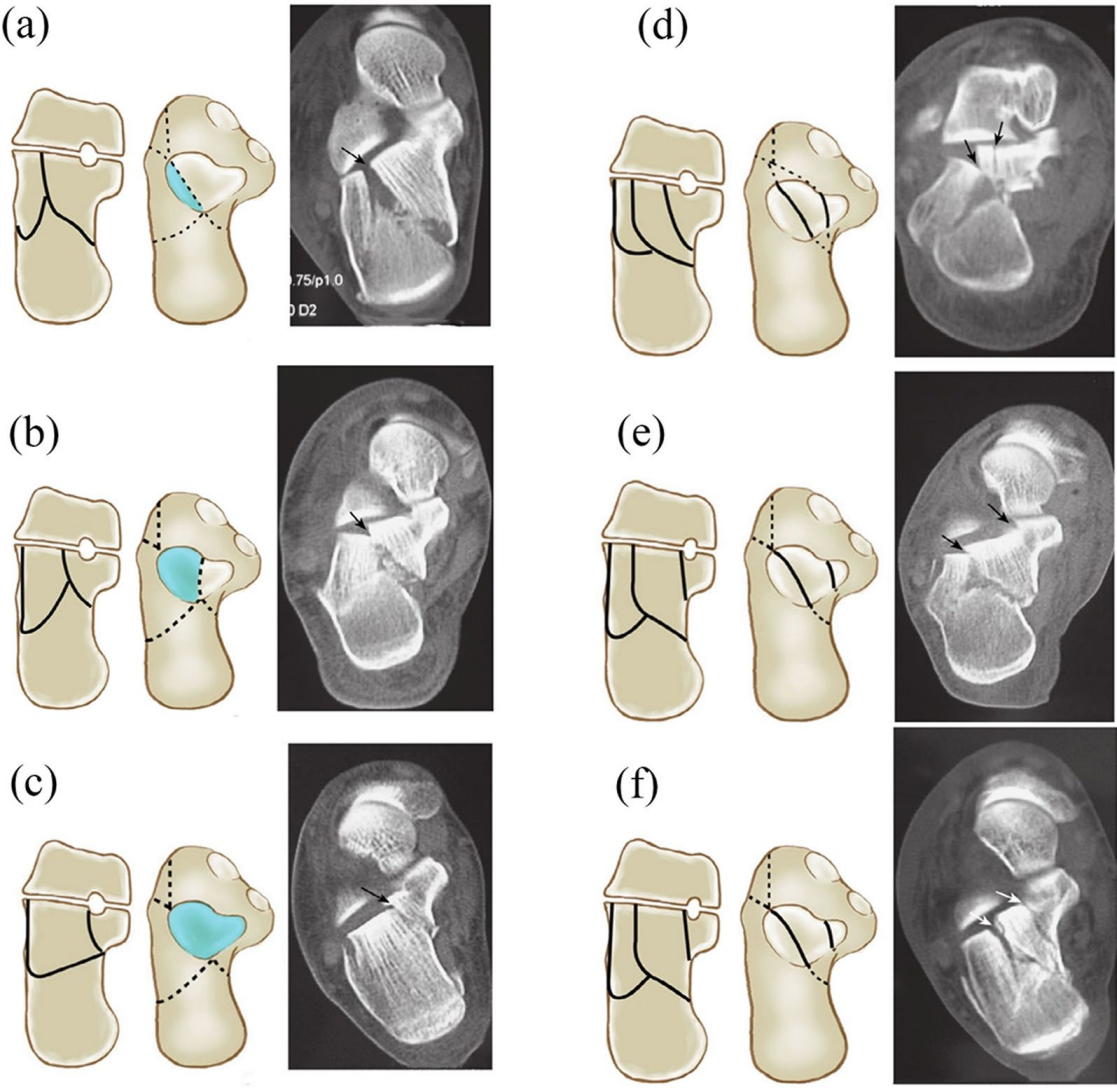
The CT images of Sanders type II and type III calcaneal fractures **a** IIa: fracture line lateral to the fracture line; **b** IIb: fracture line medial; **c** IIc: fracture line adjacent to the sustentaculum tali; and **d**, **e** and **f** three-part fracture of the posterior talar articular surface of type III: IIIab, IIIac, IIIbc

Computed tomography (CT) images of the foot and ankle of the patient (male, age 65 years, weight 64 kg) were collected from the First Affiliated Hospital of Xinxiang Medical University with a Toshiba Aquilion (Toshiba Aquilion [ONE], Toshiba, Tokyo, Japan). A total of 376 CT images were obtained from the mid tibia and fibula to the sole, with a layer thickness of 1 mm and a layer spacing of 1 mm, and were saved in DICOM format. The modelling steps are as follows: (1) The CT images were imported into Image Segmentation Software (Mimics [21.0], Materialise, Leuven, Belgium) to reconstruct the skeletal and muscular model of the foot and ankle, and optimised in Computer-aided Design Software (Geomagic Studio [2017], 3D Systems, Morrisville, USA) to remove noise and impurities, and obtain a 3D solid model with clear features and smooth surfaces; (2) the calcaneus in the foot–ankle complex was cut according to the Sanders classification to establish Sanders type II and III fracture models. The three-dimensional solid models of the plates and screws were established according to surgical standards, with the screw simplified to a solid cylinder of 3.5 mm diameter without threads, which matched with the cylindrical hole on the calcaneus [28]; and (3) the two types of fracture models were assembled with the implants to simulate the fixation of calcaneal fractures. The modelling steps are shown in Fig. 1, and the CT images of Sanders type II and type III calcaneal fractures are shown in Fig. 2 [29].

**Fig. 3:**
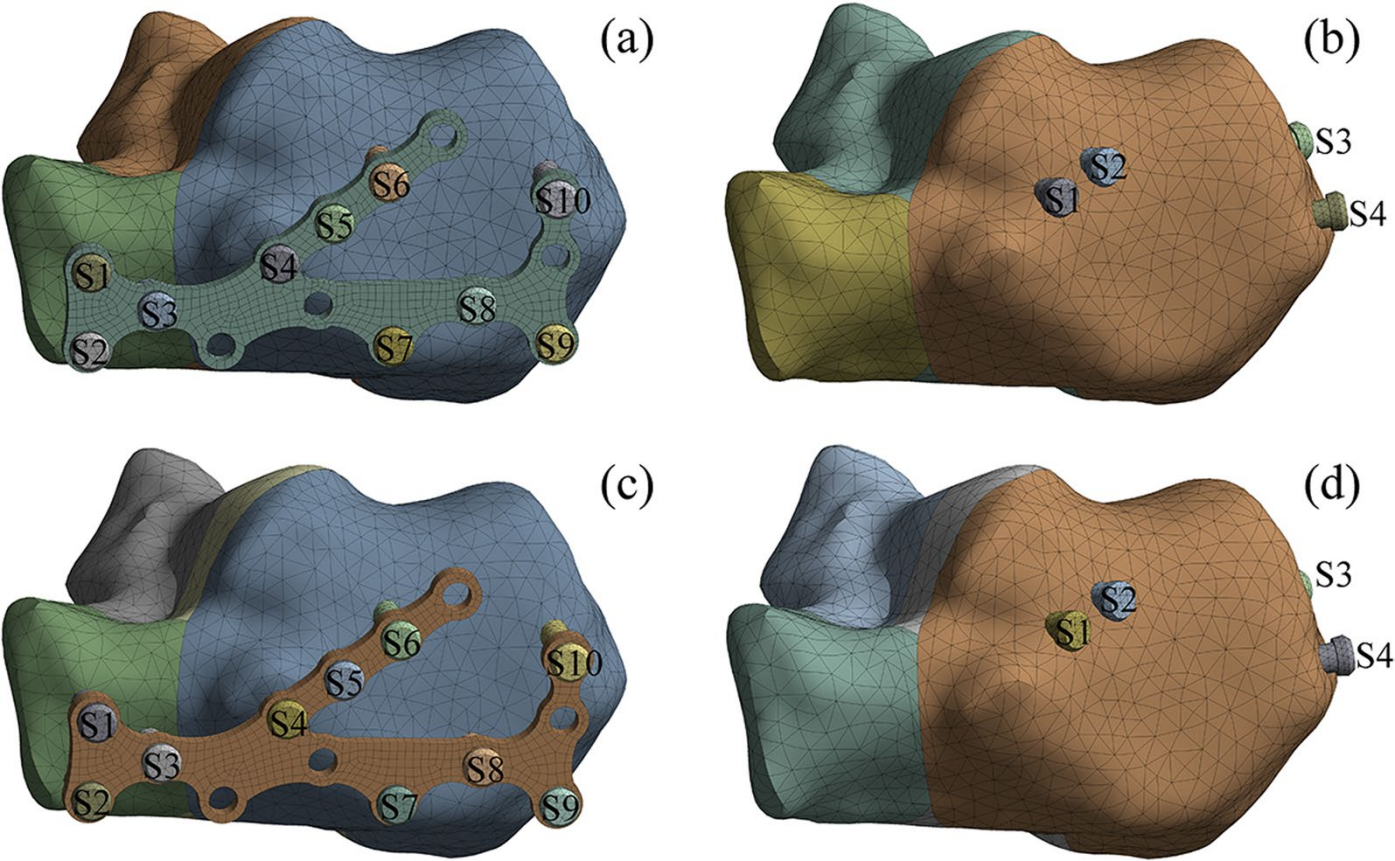
**a** Fixation of Sanders type II fracture by ORIF; **b** fixation of Sanders type II fracture by PMIF; **c** fixation of Sanders type III fracture by ORIF; and **d** fixation of Sanders type III fracture by PMIF

The bones, cartilages, soft tissue and ground support meshed with three-dimensional tetrahedral elements (C3D4). The model contains 31 major ligaments and plantar fascia such as the tibiofibular ligament, talofibular ligament, and deltoid ligament. Both the ligaments and the plantar fascia meshed with two-node truss elements. In the simulation, the principle of three-point fixation was followed; namely, the sustentaculum tali, the anterior process, and the calcaneal tuberosity were fixed by screws. The screws used in the internal fixation models of the calcaneal fracture were numbered S1-S10 as shown in Fig. 3. In the ORIF model, screws S1 and S2 fixed the anterior process, screws S5 and S6 fixed the sustentaculum tali, and screws S8, S9, and S10 fixed the calcaneal tuberosity; in the PMIF model, screws S1 and S2 fixed the sustentaculum tali, screw S3 fixed the calcaneal tuberosity, and screw S4 fixed the anterior process. The mesh convergence test was designed to test the effect of the size of the mesh elements on the calculation results. When the element size was reduced without any significant change in stress, the calculation results of the mesh used were considered to be convergent. Based on the mesh convergence test and considering the balance between the accuracy of the simulation results and the efficiency of the simulation, the mesh density of the model was determined, with a total of 248,192 elements and 142,633 nodes.

### Material properties

The different structures were assigned corresponding material properties, and in accordance with the pieces of literature [30–32], the material parameters in this paper are listed in Table 1.

**Table 1:** Material parameters

Materials	Element type	Young’s modulus (MPa)	Poisson’s ratio	Cross-sectional area(mm2)
Bone	Tetrahedron(C3D4)	7300	0.3	–
Cartilage	Tetrahedron(C3D4)	1	0.4	–
Ligament	Truss	260	0.3	18.4
Plantar fascia	Truss	350	0.4	290.7
Soft tissue	Tetrahedron(C3D4)	125	0.4	–
Titanium alloy	Tetrahedron(C3D4)	110,000	0.35	–
Ground support	Tetrahedron(C3D4)	17,000	0.1	–

### Loading and boundary conditions

**Fig. 4:**
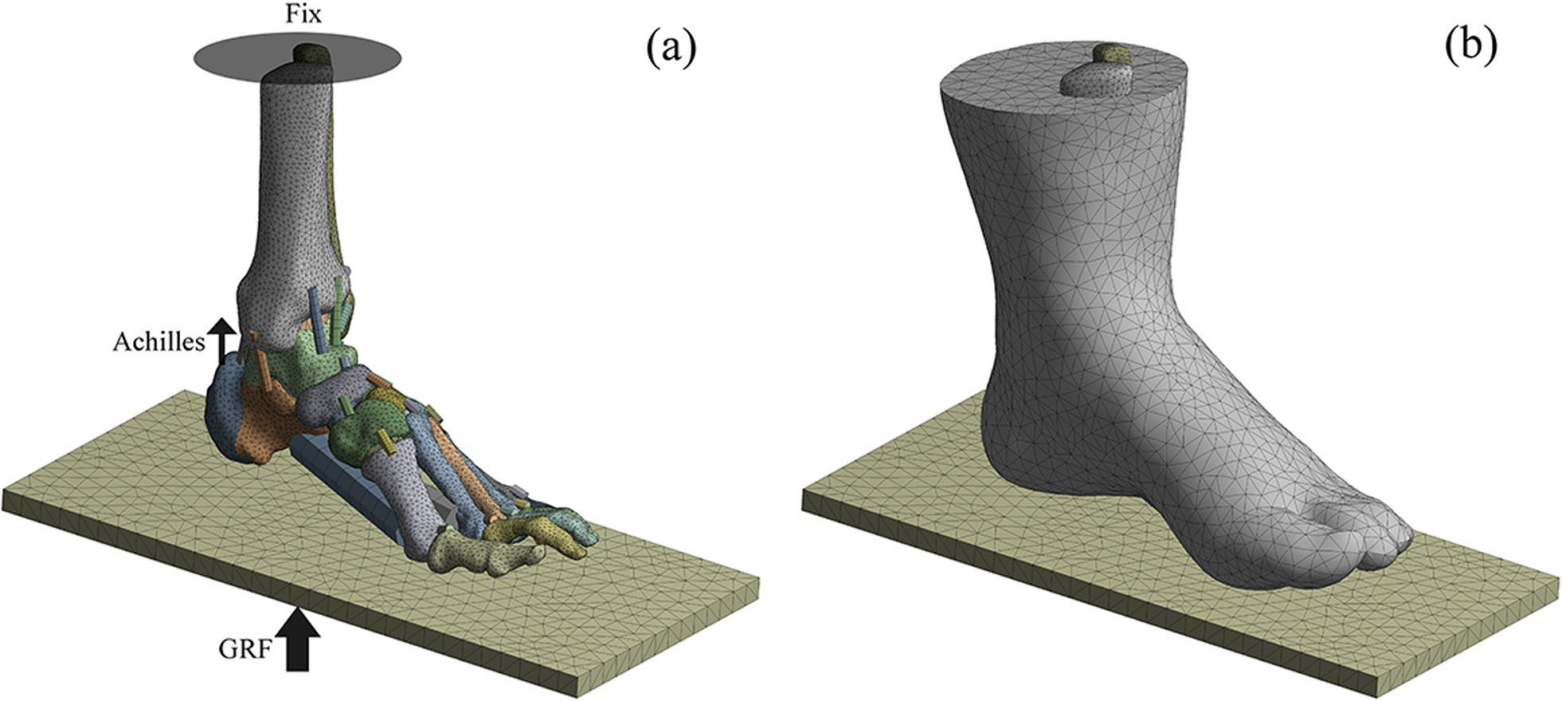
**a** The loading and boundary conditions of the model; **b** finite element model of the foot–ankle complex

The subject had a body mass of 64 kg and when standing in balance would be subjected to a ground reaction force (GRF) of 320 N on one foot, directed perpendicular to the ground and upwards. The proximal surfaces of the tibia, fibula, and soft tissue were fully constrained. The Achilles tendon force was 50% of the GRF, so there was an Achilles tendon force of 160 N at the calcaneal tuberosity, directed vertically upwards. Fracture blocks in contact with each other and between fracture blocks and screws were defined as frictional contact with a factor of 0.2, between soft tissue and ground support as frictional contact with a factor of 0.6, and between soft tissue and bones, plates, and screws as bonded contact [20]. The loading and boundary conditions of the model are shown in Fig. 4.

### Model validation

**Fig. 5:**
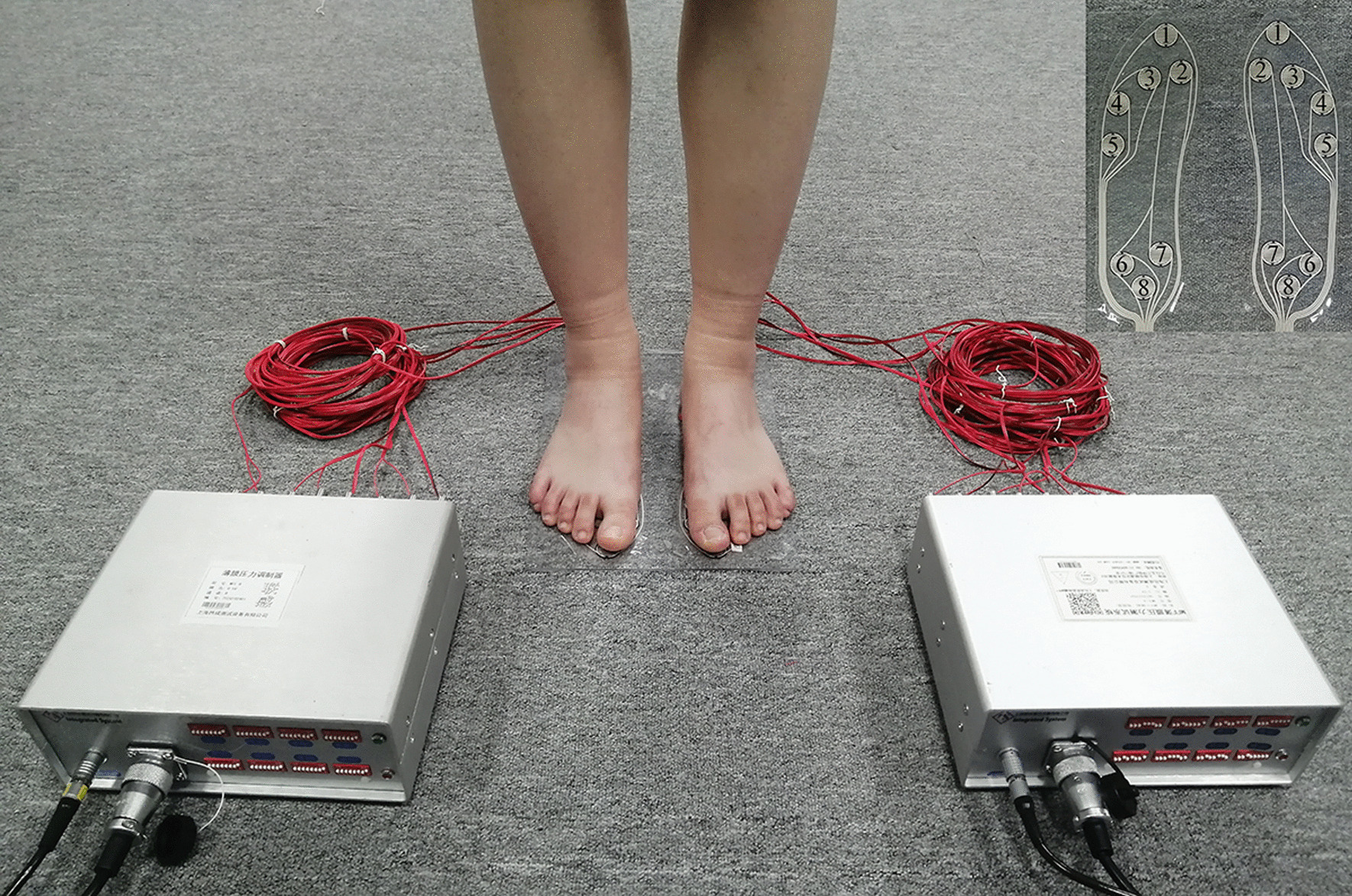
The MFF Membrane Pressure Testing System measures plantar pressure

Eight marker points 1–8 were selected on the surface of the foot-shaped membrane pressure transducer, and the plantar pressure of the subject during balanced standing was measured by the MFF Membrane Pressure Testing System (Shanghai Integrated System Technology Co., ltd, Shanghai, China) and compared with the FEA results to verify the accuracy of the model. The diameter of the membrane pressure sensors at the marked points was 2 mm and converted the output force value to a pressure value as in formula (1):

1 $$P = F/S$$

where F is the vertical force and S is the area under force. The plantar pressure was measured by the MFF Membrane Pressure Testing System as shown in Fig. 5.

## Results

### Comparison of model validation

**Fig. 6:**
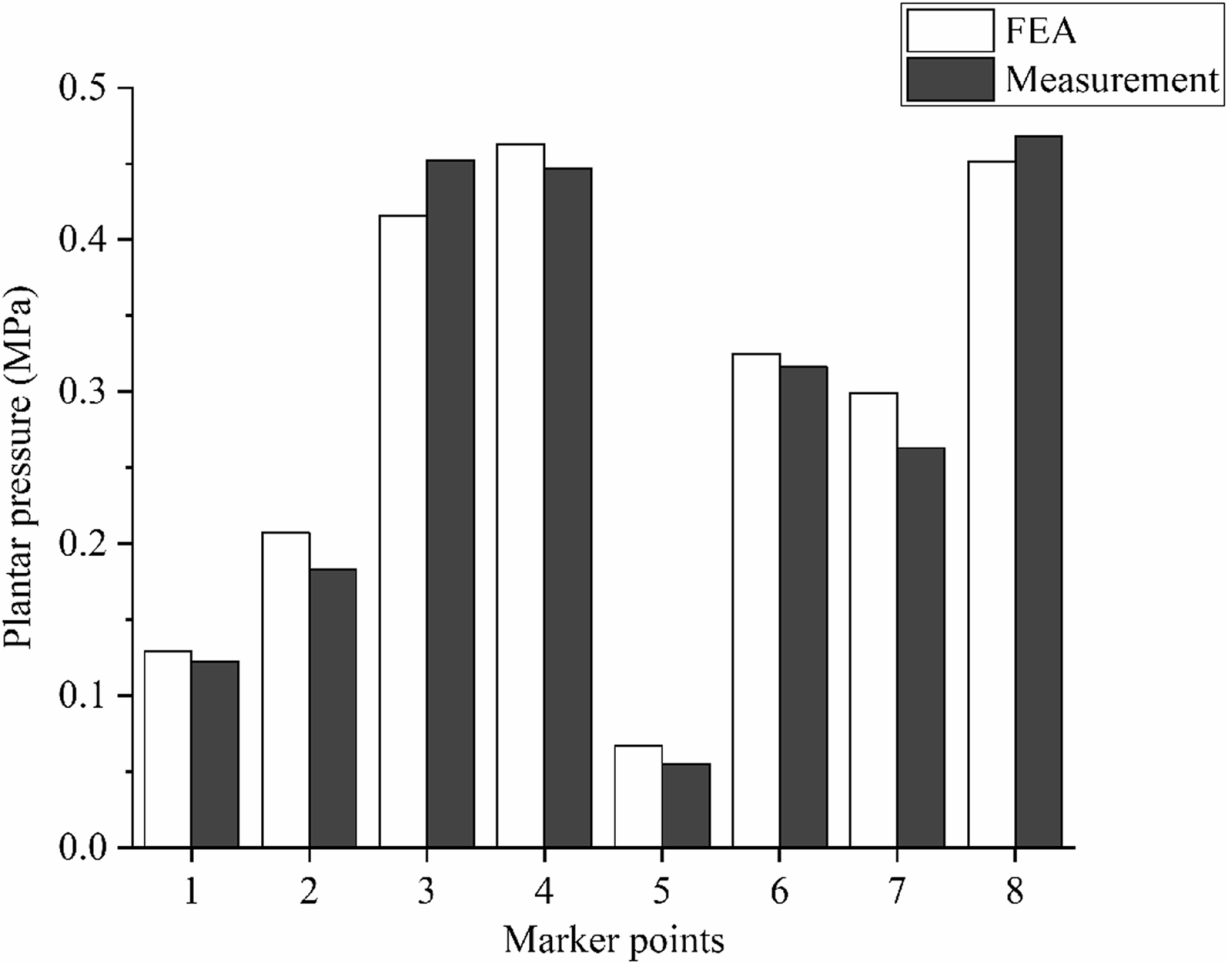
Comparison of plantar pressure distribution between FEA and measurement

The MFF Membrane Pressure Testing System and FEA results followed the same trend, with the smallest difference at marker point 6, where FEA was 2.8% higher than measurement, and the largest difference at marker point 5, where FEA was 21.8% higher than measurement. Both groups produced higher stress at marker points 3, 4 and 8 and lower stress at marker point 5. It can therefore be considered that the established finite element model of the foot–ankle complex was highly accurate and can be used in simulation studies of calcaneal fractures. The FEA and measurement results are shown in Fig. 6.

### Stress distribution

The stress distribution of the calcaneus, screws, and plates during fixation of calcaneal fractures (Sanders type II and III) using ORIF and PMIF was firstly analysed. The maximum stress on the calcaneus, screws, and plates is shown in Table 2.

**Table 2:** Maximum stress of calcaneus, screws and plates during fixation of calcaneal fractures with ORIF and PMIF (MPa)

Von Mises stress (MPa)	ORIF		PMIF	
	Type II	Type III	Type II	Type III
Calcaneus	75.85	80.54	59.66	70.02
Screw	200.56	211.59	129.61	209.46
Plate	113.88	97.06	–	–

**Fig. 7:**
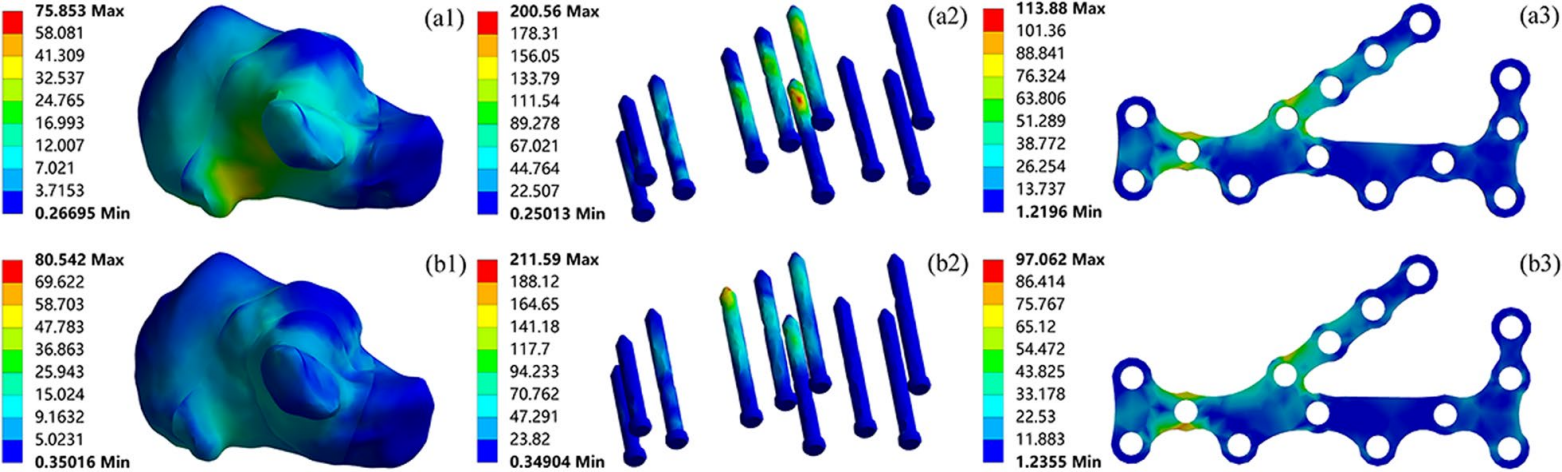
The magnitude and distribution of von Mises stress (MPa) of Sanders type II (a1-a3); Sanders type III (b1-b3) calcaneal fractures fixed by ORIF

The stress distribution of the calcaneus, screws, and plates when using ORIF to fix Sanders type II and III calcaneal fractures is shown in Fig. 7. The calcaneal stress in both types of fractures was concentrated in the medial cortex and the posterior talar articular surface, with the calcaneal stress in type II fractures occurring mainly in the medial cortex and the groove for flexor hallucis longus. The maximum stress in the calcaneus in type III fractures was greater at 80.54 MPa. In type II fractures, the screw stress was mainly concentrated in the middle of screw S7 with a maximum stress of 200.56 MPa, whereas in the type III fracture model the maximum screw stress was 211.59 MPa, mainly concentrated at the end of the sustentaculum tali screw S4. The stress distribution of the plates in type II and III fracture models was the same, mainly concentrated in the middle of the plates, with its maximum stress being larger in the type II model at 113.88 MPa.

**Fig. 8:**
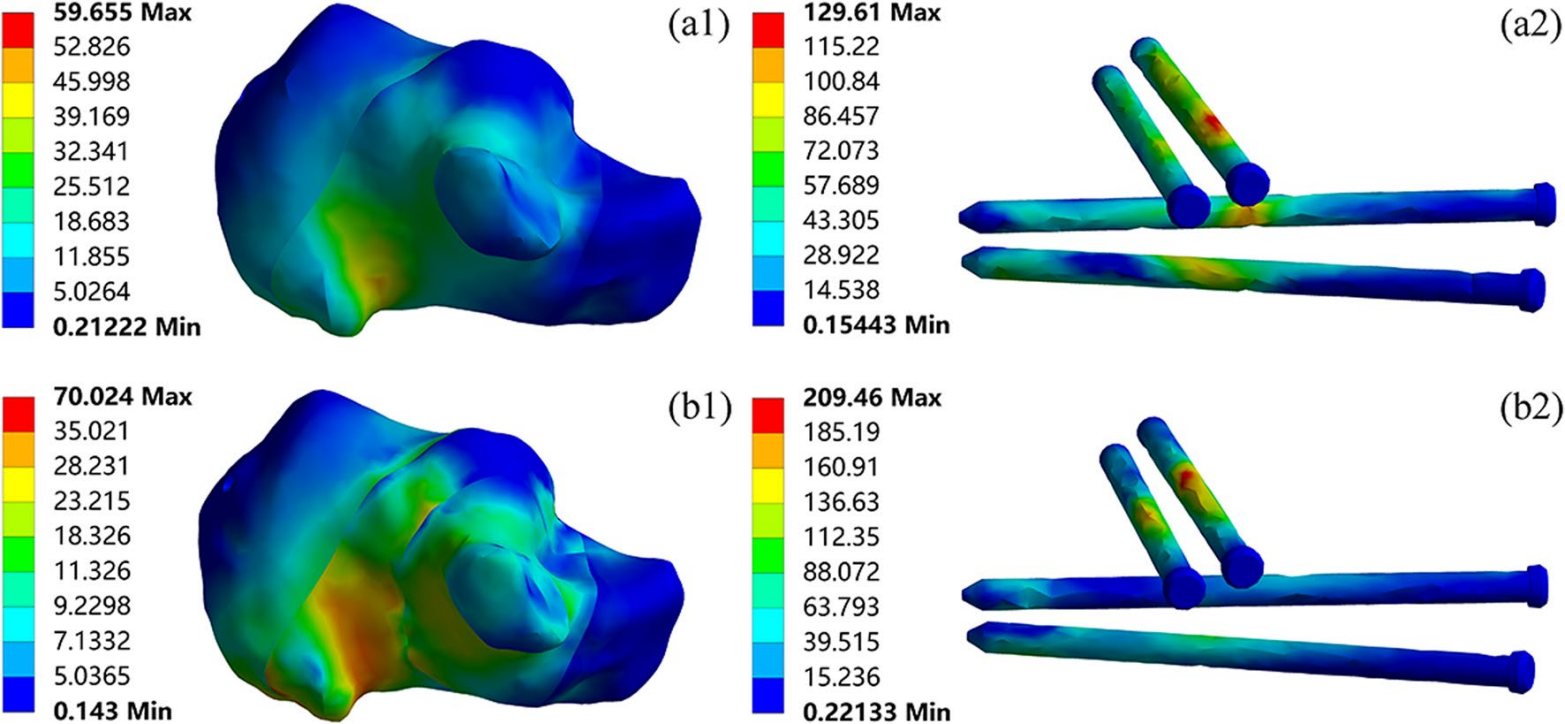
The magnitude and distribution of von Mises stress (MPa) of Sanders type II (a1-a2); Sanders type III (b1-b2) calcaneal fractures fixed by PMIF

The stress distribution of the calcaneus, screws, and plates when using PMIF to fix Sanders type II and III calcaneal fractures is shown in Fig. 8. The maximum stress in both type II and type III calcaneus was located in the medial cortex, with maximum stress of 59.66 MPa and 70.02 MPa, respectively. The overall stress on the screws was higher in the PMIF treatment of calcaneal fractures. Among them, the screw stress in type II fractures was mainly concentrated in the middle of S2, S3 and S4, with a maximum stress of 129.61 MPa; in type III fractures, the screw stress was mainly concentrated in the middle of the sustentaculum tali screws S1 and S2, with a maximum stress of 209.46 MPa.

### Displacement distribution

The displacement distribution of the calcaneus, screws, and plates during fixation of calcaneal fractures (Sanders type II and III) using ORIF and PMIF was then analysed. The maximum displacement on the calcaneus, screws, and plates is shown in Table 3.

**Table 3:** Maximum displacement of calcaneus, screws, and plates during fixation of calcaneal fractures with ORIF and PMIF (mm)

Displacement (mm)	ORIF		PMIF	
	Type II	Type III	Type II	Type III
Calcaneus	0.22	0.26	0.20	0.19
Screw	0.17	0.21	0.14	0.14
Plate	0.09	0.12	–	–

**Fig. 9:**
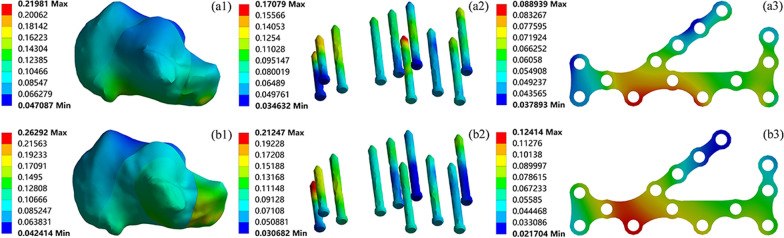
The magnitude and distribution of displacement (mm) of Sanders type II (a1-a3); Sanders type III (b1-b3) calcaneal fractures fixed by ORIF

The displacement distribution of the calcaneus, screws, and plates when using ORIF to fix Sanders type II and III calcaneal fractures is shown in Fig. 9. The displacement of the calcaneus in both fracture models was concentrated around the anterior process with a maximum displacement of 0.22 mm and 0.26 mm. The maximum displacement of the screws in type II fractures appeared at the end of S7, 0.17 mm, and in type III fractures appeared at the end of S2 fixing the anterior process, 0.21 mm. The displacement distribution of the plates in both models was similar, with the maximum displacement concentrated in the area adjacent to the posterior talar articular surface, with a maximum displacement of 0.09 mm and 0.12 mm, respectively. Simulations revealed that the maximum displacement of the calcaneus, screws, and plates in type II fracture was lower than those in type III fracture.

**Fig. 10:**
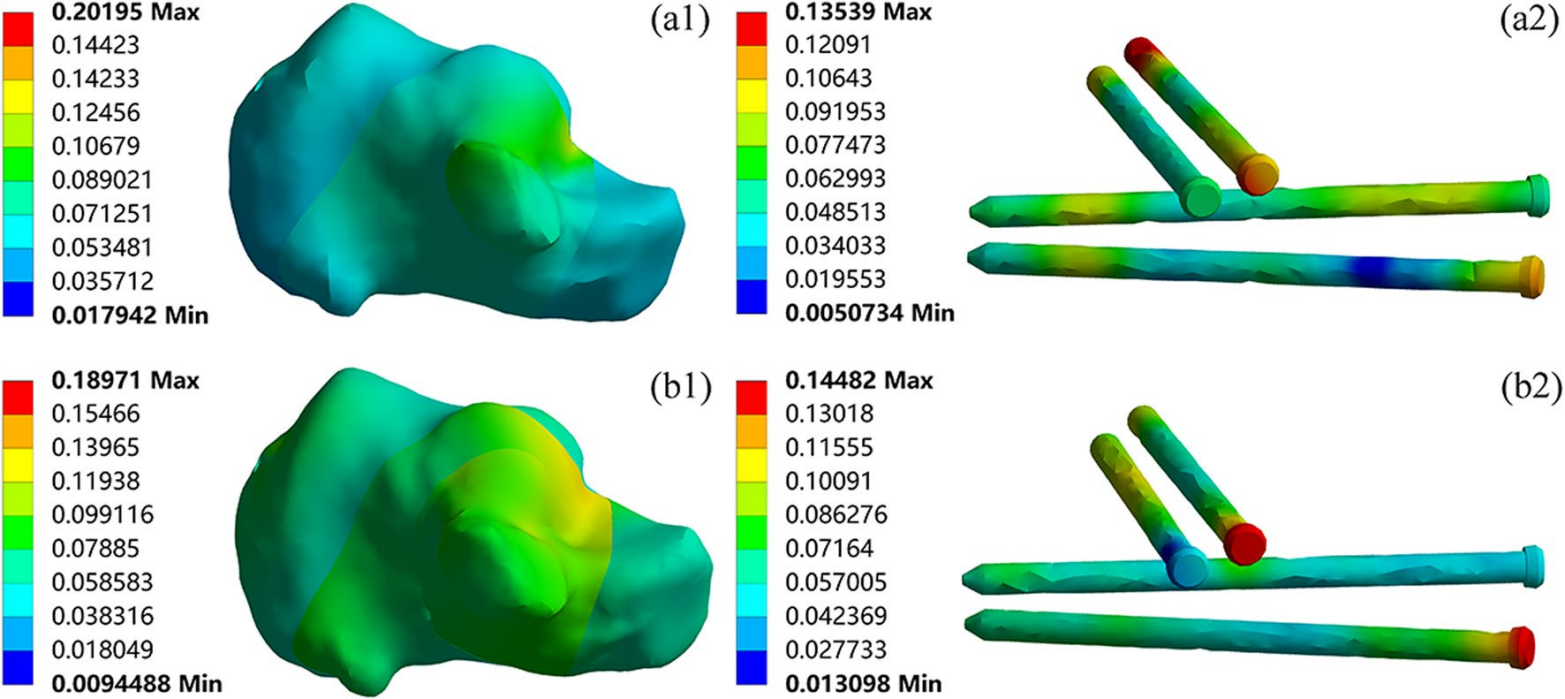
The magnitude and distribution of displacement (mm) of Sanders type II (a1-a2); Sanders type III (b1-b2) calcaneal fractures fixed by PMIF

The displacement distribution of the calcaneus, screws, and plates when using PMIF to fix Sanders type II and III calcaneal fractures is shown in Fig. 10. The maximum displacement of the calcaneus in both fracture models occurred at the posterior talar articular surface, reaching 0.20 mm and 0.19 mm. The maximum displacement on the screws in type II fractures occurred at the end of the S2, 0.14 mm; in type III fractures the maximum displacement on the screws was concentrated at the head end of S2 for fixing the sustentaculum tali and S4 for fixing the calcaneal tuberosity, 0.144 mm.

## Discussion

Minimally invasive surgery is considered to be the new trend in the treatment of calcaneal fractures. Calcaneal fractures have a high incidence and are difficult to treat clinically. This work investigated the differences in biomechanical properties between ORIF and PMIF for the treatment of calcaneal fractures (Sanders type II and type III).

Imaging data of the lower limb were collected, and a full-foot model was established. In most of the previous finite element studies of calcaneal fractures, a single calcaneus model was used without taking into account the traction of the ligaments, plantar fascia, and soft tissue on the bones [18, 33, 34]. A three-dimensional solid model of the foot–ankle complex including bones, soft tissue, cartilages, ligaments, and plantar fascia was established on this basis in this paper. Biological tissues generally exhibit nonlinear properties, but in biomechanical simulations of the bone and muscle system, the stress and deformation on the tissue are in a linear relationship when the loading is small, and the stress and strain can show a good linear elastic relationship. Therefore, the nonlinear properties of tissues can be somewhat simplified and linearly elastic material parameters can be used, which can improve the computational efficiency without affecting the computational accuracy [22, 35]. Professor Guangrong Yu [36], Department of Orthopaedic Surgery, Tongji Hospital, Tongji University School of Medicine, proposed the principle of three-point fixation of calcaneal fractures; namely, the sustentaculum tali, the anterior process, and the calcaneal tuberosity were fixed by screws. Among them, the sustentaculum tali was the most reliable fulcrum for fixing the posterior talar articular surface. It can meet the clinical need for calcaneal fracture treatment, which is of great significance in the fixation of calcaneal fractures.

The finite element results indicated that there were significant differences in the stress distribution between the two surgical approaches for the treatment of calcaneal fractures. In both the Sanders type II and III calcaneal fracture models, the calcaneal stress was distributed in the medial cortex and the posterior talar articular surface, with no significant stress concentration. This was consistent with the conclusion that the posterior talar articular surface was the main weight-bearing site. The maximum stress on the calcaneus was higher in Sanders type III than in Sanders type II for the same surgical approach. The main reason for this difference was that Sanders type III was a three-part fracture of the posterior talar articular surface, whereas Sanders type II was a two-part fracture of the posterior talar articular surface. In type III fractures, the calcaneus was more comminuted, so the calcaneus would be more stressed by the pulling forces of the plantar fascia and Achilles tendon. The maximum stress on the screws in type III fractures was higher than in type II fractures in both the ORIF and PMIF groups for the same surgical approach. In the ORIF group, the screws directly below the posterior talar articular surface were subjected to greater stress in the type II model and the sustentaculum tali screws in the type III model. The maximum stress in the PMIF group was concentrated in the sustentaculum tali screws, which was caused by the concentration of the fracture lines in the type III model from the middle of the posterior talar articular surface to the sustentaculum tali. The stress distribution of the plates was similar in type II and III fracture models in the ORIF group, suggesting that the different fracture patterns had less influence on the stress distribution of the plates. And there was no obvious stress concentration to avoid breakage of the plates due to stress fatigue.

In terms of stability of the fixation system, the PMIF achieved better stability after calcaneal fracture surgery. The maximum displacement of the calcaneus in both type II and III fracture models in the ORIF group was concentrated in the anterior process, whereas the maximum displacement in the PMIF group was concentrated in the posterior talar articular surface. This difference was related to the forces and fixation on the calcaneus in the foot–ankle composite model. The ORIF group relied on three screws for fixation of the posterior talar articular surface, with two screws driven from the outside into the inside of the anterior process. In the PMIF group, two screws were used to fix the posterior talar articular surface and the sustentaculum tali, and two screws were inserted from the end of the calcaneal tuberosity to fix the tuberosity and the anterior process. The calcaneal tuberosity screws, sustentaculum tali screws, and anterior process screws maintained the length, width and height of the calcaneus, respectively. The maximum displacement on the screws in both models was concentrated in the sustentaculum tali screws and the anterior process screws, with the maximum displacement on the screws in the ORIF group being less than 0.22 mm and in the PMIF group being less than 0.15 mm. The maximum displacement of the plates in the ORIF group appeared in the middle of the plates, which was lower than 0.13 mm, providing sufficient stability to the fixation system. Compared to ORIF, PMIF offered better clinical results for the safety and stability of the fixation system and reduced post-operative complications.

Because the stiffness of the implants (plates and screws) was much greater than the stiffness of the bone, the implants were subjected to more loading and the bone to less, thus predisposing the patient to stress shielding effect after surgery. In clinical practice, it was found that during the rehabilitation phase after internal fixation, the bone tissue was not mechanically stimulated enough due to a prolonged low-stress environment, which could easily lead to osteoporosis and secondary fractures. Resorbable implants provided adequate fixation in the initial post-operative period and gradually degraded as the bone tissue continued to heal, eliminating the need for re-surgical removal. At this stage, the challenge of reconciling the rate of implant degradation with the rate of bone healing is an urgent one.

In summary, the stress and displacement of the implants were within the permissible stress and elastic deformation. The maximum stress and displacement of the calcaneus and the implants were slightly lower in the PMIF group than in the ORIF group when fixing Sanders type II and III calcaneal fractures. Fatigue injuries are one of the main causes of fractures and implant breakage. When fatigue loading occurs, localised structural damage to the calcaneus and implants can occur. When local stresses exceed the threshold, cracks will develop at the stress concentrations, resulting in stress fractures of the calcaneus and breakage of the implants. Early post-operative weight bearing should be avoided as this may lead to loosening and breakage of the implants and displacement of the fracture fragments. Appropriate functional exercises can be performed under medical supervision. The present study still has some limitations.

1. Model: the model still needs further refinement, the cortical and cancellous bones were not segmented in this study’s model, the mechanical properties of tissues such as nerves and blood vessels were not considered, and for the time being, isotropic linear elastic material was used to simulate soft tissue and the mechanical properties of muscles were simplified;
2. Boundary conditions: static studies of balanced standing have been carried out, and complex dynamic properties (e.g. normal walking, running, impact, loading) have not been studied. The complete gait cycle consists of a stance phase and a swing phase, with the first contact of the heel with the ground being the start of the stance phase. During the stance phase, the foot changes from plantar flexion to dorsiflexion and the weight-loading area shifts from the heel to the whole foot. During heel release, the loading is distributed to the metatarsals and transferred to the phalanxes. Ankle reaction force varies with the cadence. It can reach a peak of 3–5 times body weight when walking and up to 13 times body weight when running.
3. Model validation: the measurement and FEA results have some inaccuracies due to experimental conditions and discrepancies in the model building;
4. Simulation: the contradiction between computational accuracy and efficiency has always existed. This study took into account accuracy and efficiency to the maximum extent possible under the available computing conditions. The selection of simulation parameters relied on previous studies and the actual situation of the model to obtain, and there was still some shortage in parameter optimisation. However, according to the clinical analysis, the simulation mainly plays the role of pre-assistance and subsequent validation and cannot replace clinical practice.

## Conclusions

This work investigated the differences in biomechanical properties between ORIF and PMIF for the treatment of calcaneal fractures (Sanders type II and type III). Numerical simulation results showed that the maximum stress and displacement of the calcaneus were reduced by 21.34% and 9.09%, respectively, when PMIF was applied to type II fractures compared to ORIF. The maximum stress and displacement of the calcaneus were reduced by 13.06% and 26.92%, respectively, when PMIF was applied to type III fractures compared to ORIF. The PMIF allows for better safety and stability of the fixation system, which can meet the clinical needs of calcaneal fracture surgery and post-operative functional exercise. ORIF requires extensive stripping of soft tissue and therefore has a high post-operative complication rate. The PMIF technique is rapidly developing and becoming more mature, reducing the risk of post-operative complications, in line with the modern concept of minimally invasive treatment, and deserves to be vigorously promoted in clinical practice. Follow-up research can be carried out in the following directions.

1. Establish a more realistic model. Follow-up studies follow the anatomy of the foot and ankle, and the role of the position of muscle attachment to bone is considered;
2. Accurate boundary conditions for the simulation model. In addition to simulating static balanced standing conditions, the biomechanical properties of calcaneal fractures in gait, running, impact, and loading scenarios still need to be explored for the different needs that may be encountered in the clinic;
3. Conduct more in-depth biomechanical experiments. FEA can simulate the distribution of stress and displacement within the tissue under different working conditions, but there are still differences between the calculated and actual stress conditions on the body. More in-depth biomechanical experiments can be conducted to verify and optimise the simulation model.

The Research Ethics Committee of Xinxiang Medical University approved the study (XYLL-20220211) in accordance with the Helsinki Declaration of 1975. Informed consent had been obtained from the patient, and an informed consent form had been signed.

## Data Availability

The datasets used and/or analysed during the current study are available from the corresponding author on reasonable request.
